# Within‐Night Variation in Predictor Importance Highlights Dynamic Nature of Bird Migration

**DOI:** 10.1111/ele.70422

**Published:** 2026-06-10

**Authors:** Miguel F. Jimenez, Ali Khalighifar, Kyle G. Horton

**Affiliations:** ^1^ Department of Fish, Wildlife, and Conservation Biology Colorado State University Fort Collins Colorado USA; ^2^ Department of Conservation and Science Lincoln Park Zoo, Urban Wildlife Institute Chicago Illinois USA; ^3^ Center for Bioimage Informatics St. Jude Children’s Research Hospital Memphis Tennessee USA; ^4^ Department of Forestry and Natural Resources Purdue University West Lafayette Indiana USA

**Keywords:** aeroecology, bird migration, ecological forecasting, radar ornithology, remote sensing

## Abstract

Ecological forecasting is increasingly important for conservation. Predicting nocturnal bird migration events is a promising vehicle for forecasts but isn't often explored at fine temporal scales. We use weather surveillance radar to examine dynamic drivers of migration in 2‐h periods throughout a night. We assess the relative importance of terrestrial, atmospheric and sampling predictors (which relate to radar position and scan timing) across spring and fall. Atmospheric conditions were consistently strong predictors. In contrast, terrestrial predictors contributed relatively little to explaining variation in activity. Sampling variables, such as time after sunset, varied in importance, with the highest influence shortly after sunset. We highlight the temporal variability in predictors of migration, emphasising it as a dynamic process, involving continuous decisions and adjustments rather than following fixed routes. We underscore the value of radar for capturing transitions between habitats while revealing key limitations and opportunities for understanding fine‐scale migratory behaviour.

## Graphical abstract

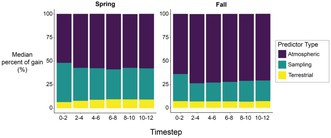

## 1 Introduction

Ecological forecasting, or the process of applying our understanding of an ecological process to predict its state into unsampled time or space, is an increasingly feasible and useful tool for advancing ecological theory and informing conservation action (Boult 2023; Lewis et al. 2023). By linking models of ecological dynamics with real‐world data, forecasting allows researchers to anticipate how populations, communities and ecosystems will respond to ongoing environmental change. As ecological pressures from climate change, habitat loss and anthropogenic drivers accelerate, our ability to make reliable predictions has become increasingly valuable. In effect, ecological forecasts can serve as a crucial bridge between science and decision‐making, informing flexible management decisions based on the best available information to mitigate risks and guide conservation priorities (Tulloch et al. 2020). Recent efforts have stressed the importance of not only improving the accuracy and scope of forecasts but also refining their temporal resolution. Developing iterative, near‐term forecasts, which are updated regularly as new data become available, represents a promising frontier for ecology and conservation science (Dietze et al. 2018). These short‐term, continually refined forecasts can help detect emerging ecological changes and support adaptive management in changing environments. Consequently, ecological forecasting stands to transform how we study, understand and manage natural systems under conditions of unprecedented change.

The study of nocturnally migrating birds is an area of research that has simultaneously served as a focal point for forecasting and a promising field for pushing it forward (Bauer et al. 2019). As part of a growing recognition that airspace represents a vast, understudied habitat that plays a vital role in many ecological processes (Diehl 2013), there have been substantial efforts to develop systems that predict the spatial–temporal patterns of birds using airspace to migrate between breeding and non‐breeding ranges (Lippert et al. 2022; Van Doren and Horton 2018). These forecasting efforts not only further the integration of ecological theory with big data approaches, such as machine learning (Jimenez, Khalighifar, et al. 2025; Kranstauber et al. 2022), but have also become crucial in guiding the management of dynamic conservation threats, such as artificial lights at night (Burt et al. 2023), bird‐aircraft collisions (Van Gasteren et al. 2019) and wind turbines (Bradarić et al. 2024). Thus, bird migration forecasts sit at the intersection of novel ecological research and innovative conservation action.

Bird migration is a large‐scale ecological process that relies heavily on airspace and terrestrial habitat, and decades of work highlight migration as a process driven by multiple, nested temporal and spatial factors (Buler et al. 2007; Van Belle et al. 2007). Migration is highly seasonal, as birds synchronise their movements to phenological resource availability during the spring and fall (Somveille et al. 2015). Within a season, the timing of migration events has long been known to be driven by favourable atmospheric conditions, such as winds that assist migratory flights (Alerstam 1972; Gauthreaux 1971). Indeed, meteorological conditions including wind, precipitation, atmospheric pressure and air temperature have proven to reliably top predictors of migration intensity (Cooper et al. 2023b; Kranstauber et al. 2022; Shamoun‐Baranes et al. 2017). Additionally, birds rely on terrestrial habitat for stopover between migratory flights, which influences the spatial distribution of where they take off and land. Terrestrial habitat serves many functions during migratory stopover including allowing for physiological recovery and refuelling from flights, providing areas where birds can avoid adverse weather conditions, and reducing predation risk (Schmaljohann et al. 2022). These benefits create strong selective pressures on birds to select stopover habitat that provides such resources (Bayly et al. 2013; Gómez et al. 2017). In practice, migrating birds regularly transition between terrestrial and airspace habitats, frequently making decisions about their relative use of each. Further, both habitats can interact in ways that influence that decision‐making. For example, the underlying topography nocturnal migrants fly over can influence their nightly timing of migration, with higher fluctuations in migration activity along coastlines relative to inland activity (Kranstauber et al. 2023). Birds flying over water reorient towards land as dawn nears (Archibald et al. 2017). Over‐water flights are energetically costly to birds due to their duration and limited opportunities to stop, causing dense stopover concentrations along large water bodies, such as the Gulf of Mexico and the Great Lakes (Bayly et al. 2018; Deppe and Rotenberry 2005; Dunn 2001), where migrants wait for favourable atmospheric conditions before advancing their aerial migration (Nourani et al. 2017). Taken together, prevailing research characterises bird migration as a highly dynamic process, whereby birds continuously alter their behaviour in response to variables across multiple, interacting scales.

Despite this, bird migration forecast systems have historically overlooked the dynamics within a given night. Often, forecasting models focus on the peak migration period 2–4 h after local sunset or evaluate model predictions across broader temporal scales, like nightly or seasonal variation (Horton et al. 2021; Jimenez, Khalighifar, et al. 2025; Van Doren and Horton 2018). Yet, a stronger understanding of the degree to which the relative importance of predictor variables changes within a given night could have important theoretical and applied implications. Models that predict migratory movements in unsampled space across a given night (Lippert et al. 2022), for example, may benefit from an enhanced knowledge of fluctuating associations. More broadly, as near‐term forecasting becomes increasingly integrated into avian conservation (Dietze et al. 2018), identifying the most informative predictors across the night may help guide and improve their development. That is, we understand bird migration as a dynamic process and forecasts that capture this temporal fluidity are likely to be more ecologically meaningful and informative for management applications.

Large‐scale radar systems have played a crucial role in providing data for migratory bird forecasts (Lippert et al. 2022). The Next Generation Weather Radar (NEXRAD) is one such system that has been used to characterise macroecosystem airspace use across the continental United States (Kelly and Horton 2016), where an estimated 70% of species are migratory (Horton et al. 2019). Unlike approaches that focus on individual movement, such as biologging (Scarpignato et al. 2023), weather surveillance radar systems like NEXRAD operate at extensive spatial scales, offering the unique ability to holistically quantify migration activity across large volumes of airspace habitat. Moreover, NEXRAD stations scan continuously, providing data on a 5–10‐min temporal scale. Together, these advantages make it an ideal system for ecological forecasting (Horton et al. 2021; Van Doren and Horton 2018) and studying the connections between terrestrial and aerial drivers of migration decisions (Clipp et al. 2020; Cohen et al. 2021; Guo et al. 2024). This presents a unique opportunity to develop forecasts with the explicit goal of capturing changes in predictors over time, including transitions between terrestrial and aerial habitats.

In this study, we employed the NEXRAD system to assess within‐night changes in the relative importance of predictors of migratory bird airspace use. Specifically, our objective was to estimate the relative strength of terrestrial, atmospheric and sampling variables in predicting bird migration activity to identify (1) patterns across a given night and (2) differences in those patterns between seasons. Based on existing literature, we predicted that terrestrial predictors would be most important in the hours surrounding local sunset and sunrise. This period is typically when nocturnally migrating birds are departing from or landing on terrestrial landscapes, and we suspected migration activity in those hours may benefit most from the inclusion of terrestrial variables. Conversely, we predicted that atmospheric predictors would be most important in the middle of each night, as that is generally the period when birds are actively navigating in broad front flights through the airspace.

## 2 Methods

We developed supervised machine learning models and used them to predict within‐night migration activity across the continental United States. We then used those models to determine the importance of variables throughout different portions of the night. Here, we describe the methods we used for data acquisition, processing, and our approach to model training, performance evaluation, and predictor importance determination.

### 2.1 Weather Surveillance Radar Data

As a measure of migration activity, we used radar data collected through the NEXRAD network, which is operated by the National Weather Service, the Federal Aviation Administration and the U.S. Air Force. The NEXRAD network consists of Doppler weather radars (WSR‐88D) that scan 360° at 0.5° azimuthal intervals (e.g., 720 azimuths) and multiple elevation angles (e.g., 0.5°, 1.5°, …, 4.5°) to monitor the airspace every 5–10 min. We downloaded level‐II radar scans which are accessible through Amazon Web Services (https://registry.opendata.aws/noaa‐nexrad/), in 30‐min intervals, or the closest available scan, between 0 and 12 h after local sunset from 2012 to 2021 (Spring: 1 March–15 June; Fall: 1 August—15 November) to capture all hours of possible nocturnal migration. Within these periods, we downloaded polar volumes, or a collection of azimuthal scans, for all 143 stations in the continental United States. From NEXRAD data, we specifically focused on reflectivity, which is commonly used as a metric of migratory bird activity.

We replicated the clutter mitigation methods of Horton et al. (2023) through the R package *bioRad* (Dokter et al. 2019) to reduce the influence of non‐avian targets. Specifically, we applied the MistNet algorithm function to filter out precipitation from polar volumes (Lin et al. 2019) and when necessary, we removed whole scans when precipitation contamination exceeded 30% between a range of 7.5–80 km. We also applied static binary clutter masks to remove topographical and ground‐based clutter. To account for the angled radar beam sampling higher altitudes with increasing range, we constructed vertical profiles of reflectivity, which integrate reflectivity from multiple tilt angle sweeps (Buler and Diehl 2009). With these vertical profiles of reflectivity, we range corrected planned position indicators (PPIs) using the integrate_to_ppi function in bioRad (Kranstauber et al. 2020). This range correction reduces the bias associated with angled radar beams, which sample at increasing height above ground level with increasing distance from the radar (Jimenez, Haest, et al. 2025). We used this function to calculate the expected reflectivity at each pixel in each PPI as weighted by the vertical profile of reflectivity and excluded individual PPI pixels that had range adjustment factors greater than 10. The result of our processing was a range‐corrected measure of vertically integrated reflectivity (cm^2^/km^2^), which we projected as a raster of within‐night bird migration intensity aggregated to ~3 km resolution. For all models, we cube‐root transformed this response variable to fit model assumptions.

### 2.2 Predictor Variable Selection

Given our goal of estimating the relative importance of different predictor variables, we sought to select a broad suite of predictor variables, some which remained static throughout the migration season (e.g., landcover), some that varied night‐to‐night (e.g., ordinal date), and some that varied within the night (e.g., wind speed). To identify these predictors, we drew heavily upon previous work, calculating atmospheric, sampling and terrestrial predictors used in previous studies from data sources available in our study system (Bradarić et al. 2024; Jimenez, Khalighifar, et al. 2025; Kranstauber et al. 2020; Van Doren and Horton 2018). We include a full list of our predictors in Table S1 and now describe the three broad classes of predictors we identified.

### 2.3 Atmospheric Predictors

We used North American Regional Reanalysis (NARR) data to characterise atmospheric conditions across the continental United States (Mesinger et al. 2006). The NARR model provides measurements of variables at 29 pressure levels every 3 h, with a 32 km resolution. We downloaded NARR data from 2012 through 2021. From this dataset, we extracted variables including air temperature (°C), geopotential height (m), zonal (U, east/west) and meridional (V, north/south) wind components (m s‐1), surface pressure (Pa), relative humidity (%), visibility (m), mean sea level pressure (Pa) and total cloud cover (%). For variables available at multiple pressure levels (air temperature and zonal and meridional wind speed), we extracted data from 1000 to 800 hPa at 50 hPa intervals.

In addition to local conditions surrounding each 2.9 km sampling pixel, we extracted atmospheric predictors from spatially distant areas. That is, for each atmospheric predictor, we calculated conditions at locations 150 km from each focal point in all cardinal directions. We included distant predictors to reflect conditions at a set range away from the focal area to capture the possibility that birds respond to environmental cues at a macroscale. This distance is within the range of average nightly flight distances of Swainson's Thrush ( *Catharus ustulatus* ) (Wikelski et al. 2003) and has been used to reflect remote conditions for migratory birds by previous studies (Horton et al. 2023; Jimenez, Khalighifar, et al. 2025; Jimenez, Haest, et al. 2025).

### 2.4 Sampling Predictors

For each radar scan, we calculated several sampling predictors that have been shown to have a relationship with NEXRAD‐derived measures of bird migration activity (Van Doren and Horton 2018). We calculated the ordinal date and time after local sunset for each NEXRAD station during which data were collected. We also estimated the distance of each pixel to the NEXRAD station collecting data and used NASA's Digital Elevation Model to calculate the elevation of each pixel at a 30 m resolution (Crippen et al. 2016).

### 2.5 Terrestrial Predictors

We calculated terrestrial predictors in three categories: land cover, vegetative phenology and artificial light at night. We used the MODIS Land Cover Type Product (MCD12Q1 v 6.0; Sulla‐Menashe et al. 2019) to estimate land cover predictors. This product includes six distinct land cover classifications at ~500 m resolution and is updated annually. We used the University of Maryland classification scheme, which provides 16 classes/types of land cover. We consolidated land cover types by combining the Cropland and Cropland/Natural Vegetation classes as cropland cover, Evergreen Needleleaf Forests, Evergreen Broadleaf Forests, Deciduous Needleleaf Forests, Deciduous Broadleaf Forests and Mixed Forests as forest cover, Closed and Open Shrublands as shrub cover, and Savannas and Woody Savannas as savanna cover. With landcover reclassified from 16 to 9 classes, we calculated percent cover for each cover type at a 2.9 km resolution.

We used the MOD13A1 v6.0 (~500 m resoltion) layer to extract enhanced vegetation index (EVI) and aggregated EVI values to 2.9 km resolution using the mean (6 × 6 cells) (Didan et al. 2015). We also calculated the standard deviation of EVI per grid cell. We used NASA's visible infrared imaging radiometer suite (VIIRS; the ‘vcm’ version of tiled monthly cloud‐free DNB composites) dataset as an index of anthropogenic light at night at a 15 arc sec (~500 m) spatial resolution (available at the Colorado School of Mines' Earth Observation Group webpage: https://eogdata.mines.edu/products/vnl/). We applied the same method as EVI to this dataset to calculate the mean and standard deviation values of radiance derived from VIIRS. In total, this process added two vegetative phenology, two artificial light at night predictors and nine landcover predictors, for a total of 13 terrestrial predictors across to be included in our forecasting model.

Similar to atmospheric predictors, for each terrestrial predictor, we calculated conditions at locations 150 km from each focal point in all cardinal directions.

### 2.6 Model Training and Performance Evaluation

We used gradient boosted trees via the *LightGBM* R package to analyse the associations between bird migration intensity and our predictor variables. We fit models for each 2‐h interval following local sunset (i.e., hours 0–2, 2–4, 4–6, 6–8, 8–10 and 10–12). Across all training locations, the mean night length for both spring and fall fell between 10 and 12 h (spring = 10.6 ± 1.1; fall = 11.9 ± 1.2), ensuring our models primarily captured nocturnal periods. To assess differences between seasons, we built separate models for spring and fall migration.

To represent spatial heterogeneity across the country while accounting for computing limitations, we subsampled the area within each NEXRAD station domain. Specifically, we randomly selected 20% (or approximately 2020 pixels) from approximately 10,000 pixels representing NEXRAD coverage and used the full 10 years of data across those pixels to build a dataset for model training, validation and holdout experiments. Within this dataset, we allocated 70% for model training, 15% for validation to tune hyperparameters, and the remaining 15% for evaluating model performance. We identified objective, learning rate, max depth, number of leaves, type of tree learner and training steps as the most important hyperparameters for model performance and used a grid search to test a range of values for each. We trained models to determine the best value for each hyperparameter (objective = regression, learning rate = 0.1, max depth = 30, number of leaves = 500, type of tree learner = serial and training steps = 10). To prevent overfitting during training of our model, we employed early stopping using the root mean square error as the monitoring metric to guide model training.

We used the coefficient of determination (*R* ^2^) values to evaluate model performance, which is a statistical measure of the proportion of variance in the dependent variable, in this case migration activity, that is predicted by independent variables. *R* ^2^ has been demonstrated to be an informative regression analysis evaluation (Chicco et al. 2021) and is commonly used as a metric of predictive performance. To compare the relative contributions across predictors, we calculated the gain‐based importance of each predictor variable. Importantly, this metric is based on the number of times a variable is selected for splitting and weighted by the squared improvement to model performance (Elith et al. 2008). Thus, if a predictor adds no unique information, its gained‐based importance remains low, partially alleviating concerns about the uneven number of variables across predictor types. To quantify changes in the relative influence of a given predictor type, we calculated the difference in its gain between two timesteps (Δgain). Finally, to assess the direction and strength of relationships between predictors and migration activity, we produced partial dependence plots, which show the estimated effect of a given variable assuming average effects of all other variables in the model.

## 3 Results

We trained and evaluated the performance of 10 models for each season per 2‐h time interval, for a total of 120 gradient boosting regression models. Each model was trained on over 13 million observations across 10 years of NEXRAD data.

### 3.1 Model Performance

**FIGURE 1:**
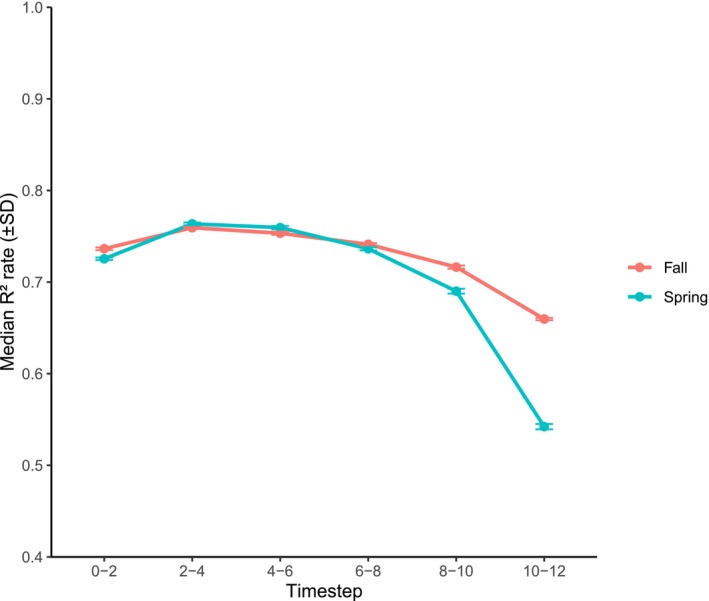
Summary of the model performance (*R* ^2^) of gradient boosted regression tree models that were used to predict bird migration activity for each timestep after sunset during spring (blue) and fall (red) migration.

The model performance for both spring and fall was stable throughout the night (spring: median *R* ^2^ = 0.73, SD ±0.08; fall: median *R* ^2^ = 0.74, SD ±0.03), with slight increases between hours two and six and a drop off after hours eight and ten. The range of model performance was consistent between seasons (spring: *R* ^2^_min_ = 0.54, *R* ^2^_max_ = 0.77; fall: *R* ^2^_min_ = 0.66, *R* ^2^_max_ = 0.76) (Figure 1).

### 3.2 Atmospheric Predictors

**FIGURE 2:**
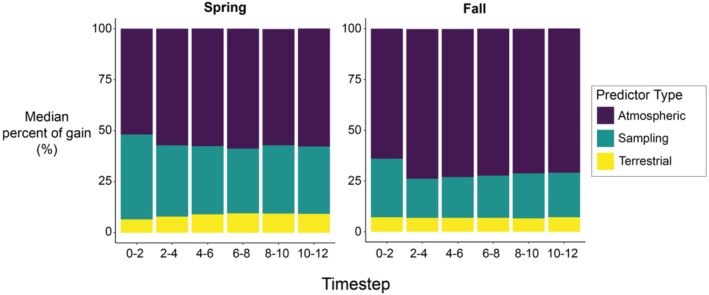
Bar chart summarising median percent of gain contributed by each predictor type (atmospheric, sampling and terrestrial) for each timestep after sunset for spring (left) and fall (right) bird migration seasons. Predictor types are denoted by different colours. Subsequent figures provide additional detail within subcategories of each predictor type and scale.

For both seasons, atmospheric predictor variables composed the majority of predictive gain across all timesteps (spring: median = 57%, SD ±3%; fall: median = 72%, SD ±4%) (Figure 2). Atmospheric importance was lowest between 0 and 2 h after sunset but increased between 2 and 4 h after sunset (spring: Δ_gain_ = 5%; fall: Δ_gain_ = 10%). During the spring, atmospheric gain peaked at 59% between 6 and 8 h after sunset. During the fall, the percentage of atmospheric gain peaked at 74% between 2 and 4 h after sunset.

**FIGURE 3:**
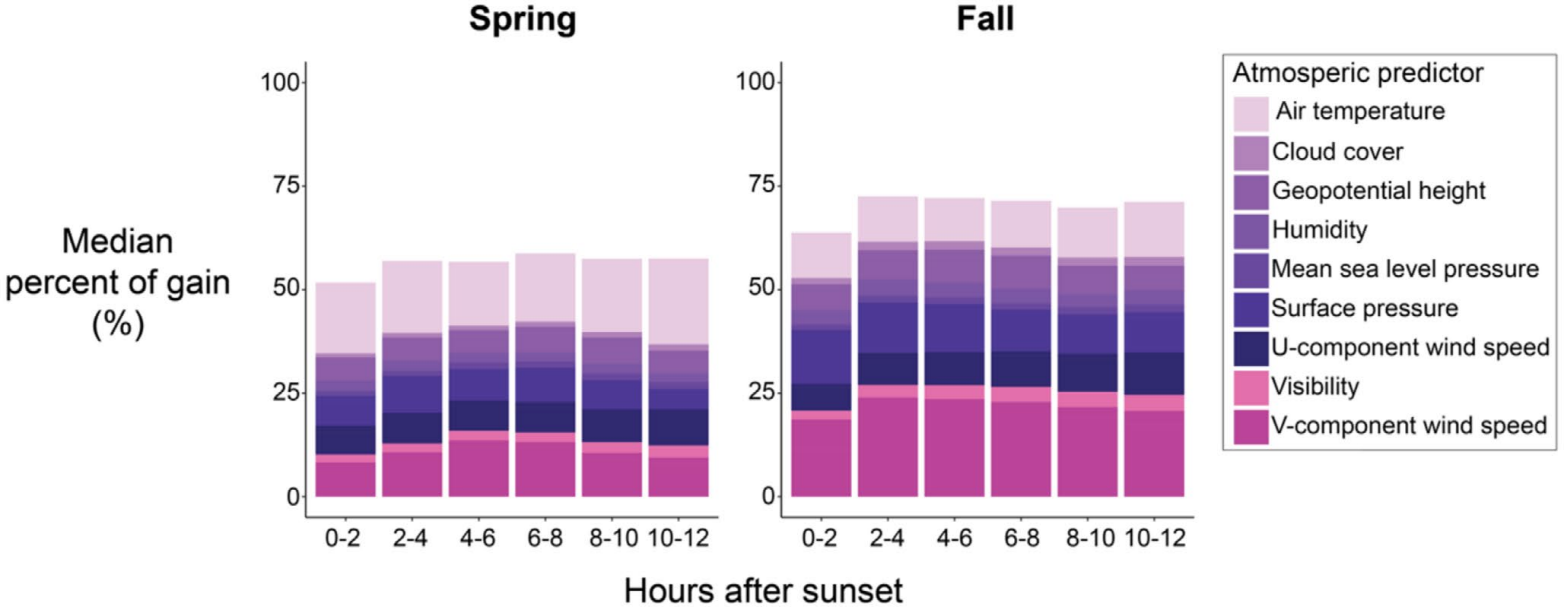
Bar chart summarising median percent of gain contributed by each atmospheric predictor in models used to predict bird migration activity throughout the night. Predictors are aggregated by different atmospheric conditions.

In spring, air temperature was consistently a top atmospheric predictor, followed by the V‐component of wind speed (north/south), surface pressure, the U‐component of wind speed (east/west) and geopotential height in varying order. In fall, the V‐component of wind speed was consistently a top atmospheric predictor, followed by surface pressure, air temperature, geopotential height and the U‐component of wind speed in varying order (Figure 3) (Figures S1 and S2).

### 3.3 Sampling Predictors

Sampling variables consistently composed the second most predictive gain (spring: median = 33%, SD ±4%; fall: median = 21%, SD ±3%) (Figure 2). Sampling gain peaked between 0 and 2 h after sunset for both seasons (spring: peak gain = 42%; fall: peak gain = 29%). In the spring, sampling gain steadily decreased between 2 and 8 h after sunset, before increasing again from 8 to 10 h after sunset (8–10 median = 33%, SD ±1%). In the fall, sampling gain was steady between 2 and 8 h after sunset, with the second highest gain between 8 and 10 h after sunset (8–10 median = 22%, SD ±1%). Across all timesteps, gain from sampling variables was higher in the spring than in the fall.

**FIGURE 4:**
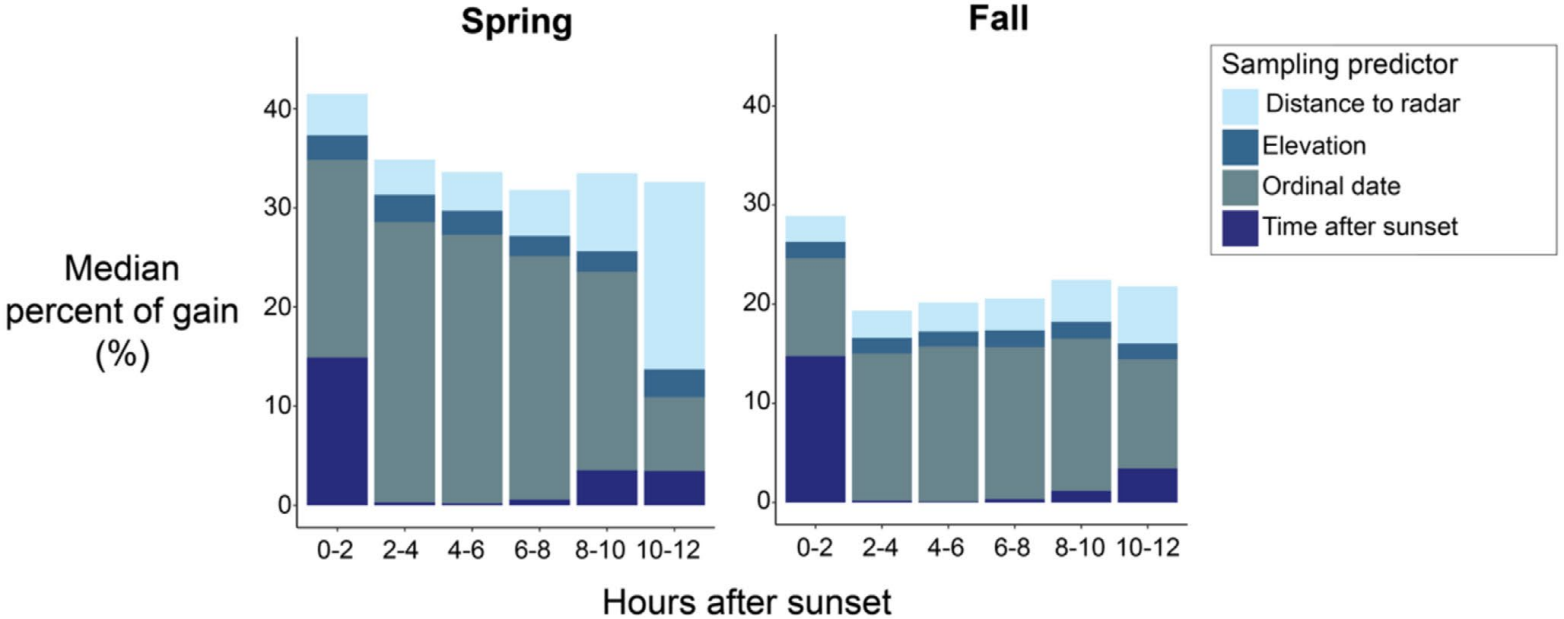
Bar chart summarising the median percent of gain contributed by each sampling predictor in models used to predict bird migration activity throughout the night. Model results are organised by spring (left) and fall (right). The percentage range is scaled down to a maximum of 45% to make small percentage contributions more visible.

Ordinal date was consistently the strongest single predictor among sampling predictors (Figure 4) and across all included variables (Figures S1 and S2). Time after sunset was most important between 0 and 2 h after sunset. Both time after sunset and distance to radar importance increased between 10 and 12 h after sunset.

### 3.4 Terrestrial Predictors

Across both seasons, terrestrial predictor variables composed a relatively small proportion of predictive gain throughout the night (spring: median = 9%, SD < 1%; fall: median = 7%, SD < 1%) (Figure S1). In spring, terrestrial importance stayed relatively consistent throughout the night but peaked later in the night between 6 and 8 h after local sunset (6–8 median = 9%, SD ±< 1%). In fall, terrestrial importance peaked between 10 and 12 h after sunset (10–12 median = 7%, SD ±< 1%) and steadily declined for the remainder of the night. Across both seasons, VIIRS nighttime light was the strongest terrestrial predictor, followed by forest cover and water bodies in varying order.

### 3.5 Predictor Scale and Direction

**FIGURE 5:**
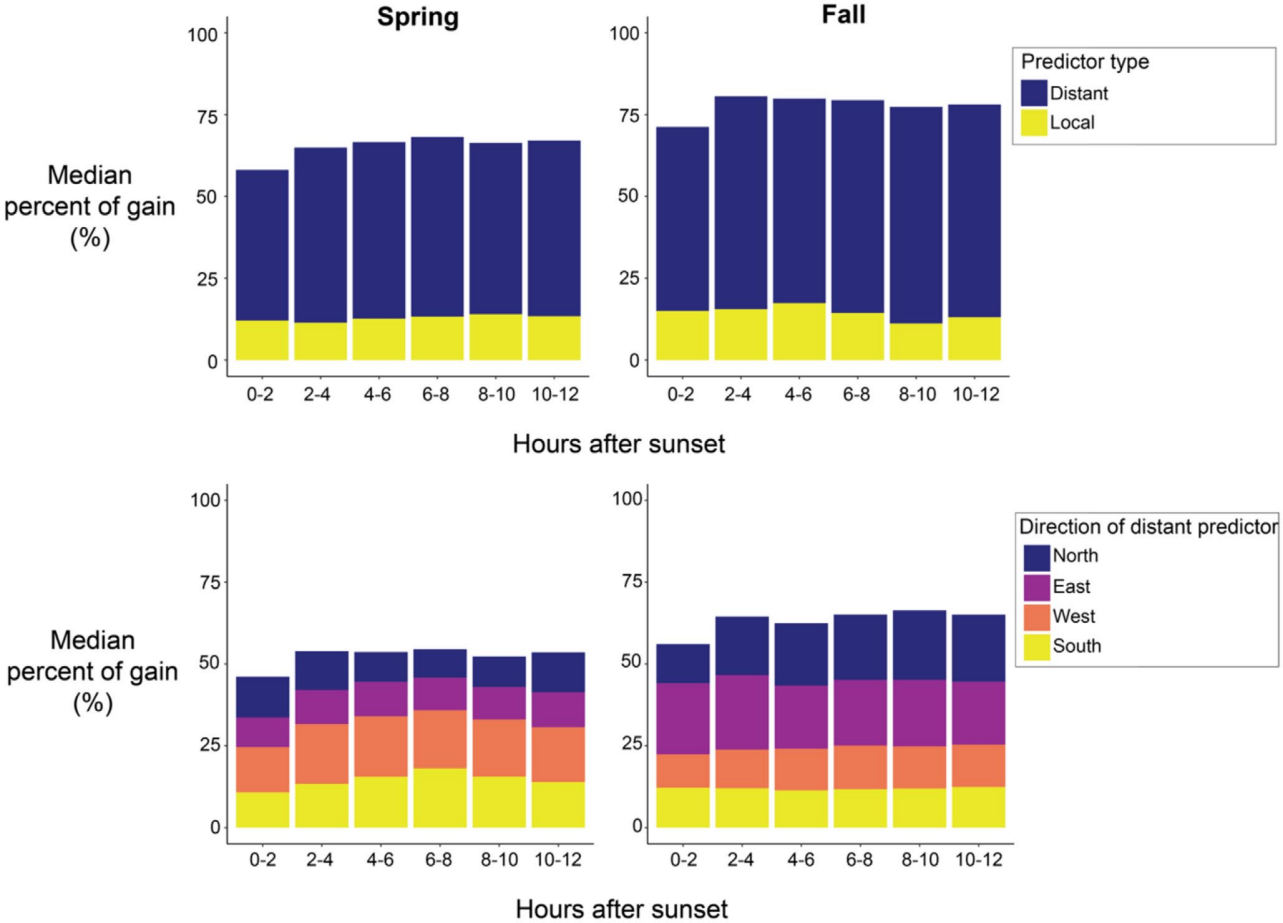
Bar chart summarising the median percent of gain contributed by distant and local spatial scale predictors (top) and the direction of distant predictors (bottom) in models used to predict bird migration activity throughout the night. Model results are organised by spring (left) and fall (right).

With respect to multi‐scale atmospheric and terrestrial predictors, spatially distant predictors composed a much larger proportion of mean gain than local predictors and their relative gains stayed stable throughout the night (distant: median = 53%, SD ±3%; local: median = 12%, SD ±< 1%) (Figure 5). North and easterly distant conditions were stronger predictors in the fall season, whereas south and westerly conditions were stronger predictors in the spring season (Figure 5).

## 4 Discussion

Our goal was to assess potential changes in the relative importance of terrestrial, atmospheric and sampling variables in predicting bird migration activity by developing separate forecasts for different timesteps within a night. While we found that atmospheric predictors were consistently the strongest predictors of activity throughout the night for both seasons, there were several aspects of our findings that were surprising. Contrary to our prediction that terrestrial variables would be most important in the beginning and end of nights, in our models they showed marginal predictive importance overall. In contrast, sampling predictors fluctuated strongly, with high importance shortly after local sunset and again before sunrise. Also unexpectedly, predictors representing environmental conditions 150 km from the focal location emerged as important drivers throughout the night. To our knowledge, our study is the first to explicitly study predictors of migratory bird activity as a dynamic process and we discuss the potential implications of our findings on future research and forecasting efforts.

Our finding that atmospheric variables were a major, consistent predictor of migration activity supports decades of research suggesting that weather conditions strongly influence avian migratory behaviour and timing (Åkesson and Hedenström 2000; Cooper et al. 2023b; Liechti 2006; McCabe et al. 2018). Few studies have explicitly compared the relative importance of different predictor types, and our study helps illuminate the drivers of bird migration through this lens. Our results are consistent with Cohen et al. (2021), who used a suite of predictors to estimate spatial drivers of stopover, finding that weather variables were strong predictors of migratory stopover. Additionally, our top atmospheric predictors (namely air temperature and wind speed components) were consistent with previous migration forecasting efforts (Van Doren and Horton 2018). Beyond this, our study design allowed us to consider how some variables may be dynamic in nature and provide additional nuance within these relationships. For example, we found some support for our prediction that atmospheric conditions would be most important in the middle of the night, as atmospheric predictive gain was highest between 2 and 8 h after sunset. This could reflect a shift in what cues birds are responding to throughout the night, such as their reorientation when approaching water bodies at dawn (Archibald et al. 2017). Overall, our results refine existing notions of how migratory birds use airspace and suggest that some aspects of this relationship change at varying temporal scales.

Contrary to our predictions, terrestrial predictors held relatively little predictive gain across the night. While we did see a very slight peak in terrestrial importance late in the night (hours 8–10) for spring and early (hours 0–2) for fall, overall terrestrial variable importance was marginal relative to atmospheric variables. Our top terrestrial predictors of VIIRS nighttime light, forest cover and water cover were similar to previous work on migration stopover (Cohen et al. 2021; Guo et al. 2023; Horton et al. 2023). This doesn't necessarily mean that terrestrial habitat isn't important, but that it isn't a central driver in predicting nightly variation in migration activity. While we are cautious not to discount the importance of terrestrial habitat on migratory behaviour (Bayly et al. 2016; Buler et al. 2007), we submit that our findings may stress the importance of including airspace in habitat when trying to understand migratory behaviour. A review of migratory stopover research, for example, highlighted remarkably inconsistent support for the relationship between terrestrial habitat quality and stopover duration (Schmaljohann et al. 2022). Our results may suggest that the migratory assistance provided by atmospheric conditions weighs heavily against the refuelling potential offered by terrestrial resources. While we recognise the importance of considering terrestrial habitat quality across the avian full annual cycle (Gunnarsson et al. 2005; Johnson 2007), we suggest that integrating the role of atmospheric conditions may provide a more holistic model for understanding avian migratory decisions and tradeoffs.

Sampling variables were the second most important predictor in our models and their relative importance showed considerable variation throughout the night. Similar to atmospheric variables, our findings are consistent with previous studies (e.g., Van Doren and Horton 2018), for example, ordinal date was a consistently strong predictor of aerial activity, which reflects the strong seasonality of migration. On the contrary, time after sunset was highly dynamic, with strong importance early in the night and much weaker importance throughout the rest of the night. We suggest that this change in importance is likely reflective of birds taking off shortly after local sunset and entering the radar beam. That is, sunset serves as a cue for nocturnal migrants to begin their flights en masse (Coppack et al. 2008; Schmaljohann et al. 2011) and, consequently, the period following sunset is when weather radar begins to detect birds aloft. However, during the middle of the night, activity is relatively steady and becomes detached from sunset timing. To this end, we also observed an increase in the importance of the time after sunset at the end of the night in the spring and, to a lesser extent, in the fall. This may be indicative of a similar, inverse sampling effect as birds land shortly after sunrise and leave the radar beam. This temporal pattern of take‐off and landing with local sunset and sunrise, respectively, has been well documented (Dinevich et al. 2003) and our findings suggest a related variability in the importance of different sampling variables throughout the night, which have implications for interpreting weather radar data and forecasting efforts.

Several aspects of our findings and limitations of our model design may help advance bird migration forecasting or serve as a foundation for future research. Most prominently, R^2^ values in both seasons dropped considerably beyond 10 h, with a particularly steep decline in spring, suggesting that model predictions are generally less reliable at the 10–12‐h timestep. It is important to note that it is unclear whether this reflects a limitation in our modelling design or an actual ecological response. On the former, we standardised our model predictors to orient around local sunset. However, our approach does not currently integrate geographic variation in night length based on local sunrise. While there is some individual variation in nightly departure timing (Müller et al. 2016), it is largely understood as an event strongly synchronised to civil dusk across nocturnal migrants (Cooper et al. 2023a). In contrast, tracking efforts have revealed high variation in migratory flight lengths (Bowlin et al. 2005; Liechti et al. 2018) and arrival timing is typically a more staggered event (Giuntini et al. 2025). Yet, in certain contexts, such as along coastlines that birds migrate over, local geography can dictate nocturnal temporal patterns (Abbott et al. 2023). Forecasting models that account for varying night length allow for quantile analyses and exploration of relationships between migration timing and habitat features, such as geographic barriers (Kranstauber et al. 2023). Future forecasting models that integrate this approach across large scales with the NEXRAD system would be a compelling research direction.

In contrast to previous forecasting efforts (Van Doren and Horton 2018), we also did not include latitude and longitude as explicit predictors. Our approach was based on the notion that distant environmental predictors inherently encode related spatial information. Surface pressure gradients, for example, reflect topographic variation. However, we interpret this as a reflection of spatially structured environmental conditions rather than a methodological confound: these variables capture the actual conditions that shape migratory behaviour, not arbitrary geographic positions. Thus, we deliberately excluded geographic coordinates from our model to preserve our ability to compare ecologically meaningful predictor types, as latitude and longitude would absorb variance across all predictor categories without providing mechanistic insight.

We were somewhat surprised to find that distant predictors, which reflected conditions 150 km from each focal area, were consistently strong predictors, as they cannot be experienced directly by migrating birds. Indeed, previous studies have highlighted the value of including remote or distant variables in forecasting models (Jimenez, Khalighifar, et al. 2025; Jimenez, Haest, et al. 2025; Kranstauber et al. 2022). Taken together, our results may be consistent with the notion that migration behaviour is largely driven by synoptic weather conditions, highlighting the predictive value of including conditions in departure locations (Manola et al. 2020). We suggest that carefully selecting predictors that best capture synoptic conditions could accelerate the development of effective near‐term predictive models using fewer predictors. Additionally, while we tested dimensions of time in space in our predictor sets, we did not explore variable buffer sizes for averaging these predictors, which could help us understand the spatial scale that terrestrial and atmospheric variables drive decisions (e.g., local vs. synoptic weather). Such efforts could inform more nuanced selection of ‘distant’ conditions that account for conditions experienced by birds based on their specific locations.

With respect to our model structure, our goal was to determine the relative importance of a suite of predictor variables in predicting bird migration activity across a given night. However, we recognise that there are fundamental differences between how these predictors were captured in our model. We based our predictions on evidence that birds make decisions about where to land based on key terrestrial habitat features, such as forest cover, at multiple spatial scales (Buler et al. 2007; Guo et al. 2023). Yet at the within‐night scale, unlike atmospheric conditions, landcover is not a dynamic predictor. We suspect that, while landcover may not be a predictor of within‐night activity, key landcovers like forest shape the baseline of migration activity levels, whereas atmospheric conditions predict deviations from that baseline. A hierarchical approach that nests aerial conditions within terrestrial cover could be a valuable next step for this work. Relatedly, while we chose to design our study at the continental scale, region‐specific models may reveal spatial variation in terrestrial and aerial importance. For instance, terrestrial habitat may be more important in areas surrounding large migration barriers, such as large bodies of water along the Gulf Coast (Deppe and Rotenberry 2005) or cropland in the midwestern United States (Guo et al. 2023). More broadly, the volume of our predictor and model list makes it difficult to concisely interpret and discuss all relationships between each individual predictor. Yet, as new forecasting designs are being developed, we encourage the use of our supplemental figures (Figures S1 and S2) and materials for informing variable selection.

Migration is the least understood portion of the avian life cycle, which is highlighted by our limited understanding of variables that best predict migratory activity within a given night. Here, we provide insights on the relative importance of terrestrial and atmospheric variables in forecasting migration activity and shed new light on how they may change over the course of a given night. Our research underlines the importance of leveraging our understanding of airspace as habitat to refine ecological forecasting and validates the value of bird migration as a system for doing so.

## Author Contributions

M.F.J., A.K. and K.G.H. conceived the research questions. K.G.H. and A.K. performed data processing. A.K. and M.F.J. performed model fitting and analysed output data. M.F.J. visualised the data and wrote the first draft of the manuscript. All authors contributed substantially to subsequent drafts and revisions.

## Funding

This work was supported by the National Aeronautics and Space Administration.

## Supporting information


**Table S1:** A list of the sampling, atmospheric and terrestrial predictor variables used to predict bird migration density with gradient boosted trees.
**Figure S1:** (Left) Top 10 predictors of spring migration as estimated by model gain, a measure of the relative contribution of the corresponding feature to the model.
**Figure S2:** (Left) Top 10 predictors of fall migration as estimated by model gain, a measure of the relative contribution of the corresponding feature to the model. (Right) Partial dependence of the predicted migration traffic (cm^2^/km^2^) on the focal predictor, averaged over the distribution of all other predictors. Grey areas indicate 95% confidence intervals.

## Data Availability

We provide our codebase and a subset of data for the year 2012 (one of 10 years included in our study), which can be used to run modelling scripts on Figshare: https://doi.org/10.6084/m9.figshare.28755569.v2 (Jimenez 2025). We also provide our codebase on Github: https://github.com/a‐khalighifar/migration_forecast/tree/main/within_night. The full dataset used was derived from the following resources available in the public domain: Amazon Web Services (https://registry.opendata.aws/noaa‐nexrad/), North American Regional Reanalysis (https://www.ncei.noaa.gov/products/weather‐climate‐models/north‐american‐regional), MODIS Land Cover Type Produce (https://modis.gsfc.nasa.gov/data/dataprod/mod12.php), MODIS Vegetation Index (https://modis.gsfc.nasa.gov/data/dataprod/mod13.php) and NASA Visible Infrared Imaging Radiometer Suite (https://eogdata.mines.edu/products/vnl/). We include the scripts used to download and process these data on GitHub.
