# Supplementary material for: Within‐Night Variation in Predictor Importance Highlights Dynamic Nature of Bird Migration
Source: Ecol Lett. 2026 Jun 10;29(6):e70422. doi: 10.1111/ele.70422 (PMC13253007; doi:10.1111/ele.70422)
Supplement: Supplementary file 1 — Table S1: A list of the sampling, atmospheric and terrestrial predictor variables used to predict bird migration density with gradient boosted trees. Figure S1: (Left) Top 10 predictors of spring migration as estimated by model gain, a measure of the relative contribution of the corresponding feature to the model. Figure S2: (Left) Top 10 predictors of fall migration as estimated by model gain, a measure of the relative contribution of the corresponding feature to the model. (Right) Partial dependence of the predicted migration traffic (cm2/km2) on the focal predictor, averaged over the distribution of all other predictors. Grey areas indicate 95% confidence intervals. [file ELE-29-0-s001.docx]

**Table S1.** A list of the sampling, atmospheric, and terrestrial predictor variables used to predict bird migration density with gradient boosted trees.

| **Predictor** | **Class of variables** | **Resolution** | **Collection metrics** | **Distant/ Cardinal directions included** |
| --- | --- | --- | --- | --- |
| Ordinal date | Sampling | - | - | No |
| Time after sunset | Sampling | - | - | No |
| Distance from radar | Sampling | - | - | No |
| Elevation | Sampling | NASADEM (30 m) | - | No |
| Air temperature | Atmospheric | NARR (32 km) | 2 m above ground, 800-1000 hPa | Yes |
| Geospatial height | Atmospheric | NARR (32 km) | Surface | Yes |
| Pressure | Atmospheric | NARR (32 km) | Surface, mean sea level | Yes |
| Relative humidity | Atmospheric | NARR (32 km) | 2 m above ground | Yes |
| Total cloud cover | Atmospheric | NARR (32 km) | Entire atmosphere | Yes |
| Visibility | Atmospheric | NARR (32 km) | Surface | Yes |
| Zonal and meridional wind speed | Atmospheric | NARR (32 km) | 10 m above ground, 800-1000 hPa | Yes |
| Enhanced Vegetation Index (EVI) | Terrestrial | MOD13A1 (500 m) | Aggregated to 2.9 km, and mean and standard deviation collected | Yes |
| Visible Infrared Imaging Radiometer Suite (VIIRS) | Terrestrial | VIIRS Nighttime lights (~500 m at the Equator) | Aggregated to 2.9 km, and mean and standard deviation collected | Yes |
| Water bodies | Terrestrial | MCD12Q1 (500 m) | Aggregated to 2.9 km, and relative percentage calculated | Yes |
| Grasslands | Terrestrial | MCD12Q1 (500 m) | Aggregated to 2.9 km, and relative percentage calculated | Yes |
| Permanent wetlands | Terrestrial | MCD12Q1 (500 m) | Aggregated to 2.9 km, and relative percentage calculated | Yes |
| Urban and Built-up Lands | Terrestrial | MCD12Q1 (500 m) | Aggregated to 2.9 km, and relative percentage calculated | Yes |
| Non-Vegetated Lands | Terrestrial | MCD12Q1 (500 m) | Aggregated to 2.9 km, and relative percentage calculated | Yes |
| Forests | Terrestrial | MCD12Q1 (500 m) | Aggregated to 2.9 km, and relative percentage calculated | Yes |
| Shrublands | Terrestrial | MCD12Q1 (500 m) | Aggregated to 2.9 km, and relative percentage calculated | Yes |
| Croplands | Terrestrial | MCD12Q1 (500 m) | Aggregated to 2.9 km, and relative percentage calculated | Yes |
| Savannas | Terrestrial | MCD12Q1 (500 m) | Aggregated to 2.9 km, and relative percentage calculated | Yes |

**Figure S1.** (Left) Top 10 predictors of spring migration as estimated by model gain, a measure of the relative contribution of the corresponding feature to the model. (Right) Partial dependence of the predicted migration traffic (cm^2^/km^2^) on the focal predictor, averaged over the distribution of all other predictors. Gray areas indicate 95% confidence intervals.

**Figure S2.** (Left) Top 10 predictors of fall migration as estimated by model gain, a measure of the relative contribution of the corresponding feature to the model. (Right) Partial dependence of the predicted migration traffic (cm^2^/km^2^) on the focal predictor, averaged over the distribution of all other predictors. Gray areas indicate 95% confidence intervals.
